# Enhanced antifungal efficacy of azole drugs through combination with usnic acid

**DOI:** 10.3389/fmicb.2026.1864975

**Published:** 2026-10-19

**Authors:** Menghua Tang, Xiaoman Yang, Lu Ge, Shuyang Xie, Yixin Zeng, Tian Chen, Heng Zhang, Yi Sun

**Affiliations:** 1 Department of Clinical Medicine, Yangtze University, Jingzhou, Hubei, China; 2 Department of Dermatology, Hubei Provincial Clinical Research Center for Diagnosis and Therapeutics of Pathogenic Fungal Infection, Jingzhou Hospital Affiliated to Yangtze University, Jingzhou, Hubei, China

**Keywords:** antifungal azoles, *Aspergillus*, posaconazole, synergy, usnic acid

## Abstract

**Introduction:**

Drug-resistant fungal infections remain a major clinical challenge, and antifungal adjuvants offer a promising strategy to restore azole efficacy. This study investigated whether usnic acid (UA), which lacks intrinsic antifungal activity, enhances the activity of itraconazole (ITR), voriconazole (VOR), posaconazole (POS), isavuconazole (ISA), and fluconazole (FLU) against clinically relevant fungal pathogens.

**Methods:**

The synergistic effects of UA combined with each azole were evaluated against clinical isolates of *Aspergillus* spp. (*n* = 64), *Candida* spp. (*n* = 24), *Exophiala dermatitidis* (*n* = 14), and *Cryptococcus neoformans* (*n* = 8) using CLSI-standardized broth microdilution and checkerboard assays. According to their clinical use, ISA was tested against molds, whereas FLU was tested against *Candida* spp. and *C. neoformans*. Interactions were classified by the fractional inhibitory concentration index as synergy (FICI ≤ 0.5), indifference (0.5 < FICI ≤ 4.0), or antagonism (FICI > 4.0). Rates were reported with 95% Wilson confidence intervals and compared using Fisher–Freeman–Halton and pairwise Fisher exact tests with Benjamini–Hochberg correction.

**Results:**

UA alone showed no antifungal activity. The overall synergy rates were 67.27% (74/110; 95% CI 58.05–75.33%) for UA+POS, 11.82% (13/110; 95% CI 7.04–19.18%) for UA+ITR, 9.09% (2/22; 95% CI 2.53–27.81%) for UA+FLU, 5.45% (6/110; 95% CI 2.52–11.39%) for UA+VOR, and 0% (0/78; 95% CI 0–4.62%) for UA+ISA. UA+POS showed a significantly higher synergy rate than each other combination after multiple-comparison correction (*P* < 0.001). UA+ITR caused no antagonism, whereas UA+POS caused antagonism in only 0.91% (1/110) of isolates. UA+VOR and UA+FLU showed marked antagonism against *C. neoformans* (100%, 8/8 and 87.5%, 7/8, respectively). UA+POS produced broad synergy against *A. terreus* and *E. dermatitidis* regardless of susceptibility, whereas activity against *A. fumigatus* was mainly restricted to susceptible isolates. Mechanistic assays showed increased reactive oxygen species levels in five of seven synergistic strains and efflux pump inhibition in four, with strains 382 and PY1b exhibiting both mechanisms.

**Discussion:**

These findings identify UA+POS as the most promising adjuvant combination, while the species- and resistance-dependent effects suggest that its activity requires careful contextual interpretation. The dual involvement of reactive oxygen species accumulation and efflux pump inhibition supports a mechanistically diverse mode of synergy. However, these results are limited to in vitro testing, and further mechanistic and in vivo studies are required to evaluate the therapeutic potential and safety of UA+POS.

## Introduction

Invasive Fungal Infections (IFIs) represent a global health challenge, causing substantial morbidity and mortality among immunocompromised patients (Denning, 2024). High-risk groups include those with neutropenia, transplant recipients, and individuals in critical care (Webb et al., 2018; Apostolopoulou et al., 2026). The epidemiological landscape of IFIs is broadening (Hoenigl et al., 2022; Salazar et al., 2022). While *Aspergillus* and *Candida* remain predominant, pathogens like *Cryptococcus neoformans* and dematiaceous fungi (e.g., *Exophiala dermatitidis*) warrant serious attention (Casalini et al., 2024). Infections by these fungi are particularly concerning due to their frequent multi-drug resistance, which drastically confines the available treatment arsenal (Fisher et al., 2022). Azole drugs, including itraconazole (ITR), voriconazole (VOR), posaconazole (POS), isavuconazole (ISA), and fluconazole (FLU), function by inhibiting lanosterol 14α-demethylase (CYP51), a key enzyme in fungal membrane ergosterol synthesis (Handelman et al., 2021; Roundtree et al., 2020). This action compromises membrane integrity, resulting in fungistatic or fungicidal effects (Carmona and Limper, 2017; Liu et al., 2018; Seyedmousavi et al., 2017; Wiederhold, 2017). As a cornerstone for preventing and treating IFIs, the widespread and often excessive use of azoles has led to the emergence and spread of resistant strains. Resistance mechanisms include CYP51 mutations, over-expression of drug efflux pumps, and biofilm formation (Price et al., 2015; Gautam et al., 2008; Parker et al., 2014). This escalating resistance problem frequently leads to clinical treatment failures, necessitating novel strategies to overcome the challenge.

Compared to the prolonged and costly process of developing novel antimicrobials, the use of antimicrobial adjuvants presents a more efficient and timely strategy (Rosas and Lithgow, 2022; Si et al., 2022). This combination approach allows for reduced dosages of the primary drug, thereby mitigating toxicity, slowing the evolution of resistance, and extending the clinical lifespan of established therapeutics (Chen et al., 2025; Liang et al., 2025; Zhang et al., 2025; Jia et al., 2023).

Natural products, valued for their diverse chemical structures and biological activities, are a vital source for discovering novel antimicrobial adjuvants (Chen et al., 2025; Liang et al., 2025; Zhang et al., 2025). Usnic acid (UA), a unique dibenzofuran derivative extracted from lichens (such as the genus *Usnea*), has a history of use in traditional medicine for its antimicrobial and anti-inflammatory properties (Sepahvand et al., 2021). Modern pharmacological studies confirm its activity against various Gram-positive bacteria, mycobacteria, and some fungi, potentially through mechanisms like membrane disruption, energy metabolism inhibition, and oxidative stress induction (Zhang et al., 2023; Bankan et al., 2021; Sieniawska et al., 2021). Of particular interest, preliminary research suggests that usnic acid can effectively inhibit bacterial efflux pumps (Bankan et al., 2021). Based on these mechanisms, usnic acid serves as a promising antimicrobial adjuvant that can restore bacterial susceptibility to various antibiotics, including peptides and aminoglycosides, by inhibiting efflux pumps or enhancing membrane permeability (Zhang et al., 2023; Bankan et al., 2021; da Costa Junior et al., 2020). However, this potential and its role as a broad-spectrum azole adjuvant in the antifungal field has not yet been systematically investigated or evaluated.

Therefore, this study was designed to systematically evaluate the potential of usnic acid as a broad-spectrum adjuvant for azole drugs. We assessed its *in vitro* synergistic effects with five key azoles (ITR, VOR, POS, ISA and FLU) against a wide range of clinical isolates, including *Aspergillus* spp., *Candida* spp., *E. dermatitidis*, and *C. neoformans*. In addition, the underlying mechanisms were preliminarily investigated. This work aims to fill a critical knowledge gap and identify novel combination therapies to address the growing problem of azole resistance.

## Materials and methods

### Fungal strains and culture conditions

Antifungal susceptibility testing was performed using the broth microdilution method in accordance with CLSI guidelines. All isolates were procured from Jingzhou Central Hospital (Hubei, China) and the Hubei Clinical Medical Center for Pathogenic Fungal Infections (Chen et al., 2025), with identity confirmed by morphological assessment and ITS rDNA sequencing. The quality control strains *Aspergillus flavus* ATCC 204304 and *Candida parapsilosis* ATCC 22019 were employed. All strains were maintained on Sabouraud Dextrose Agar (SDA) and incubated at 35 °C.

### Chemicals and reagents

The antifungal agents and solvents used in this study were commercially sourced. The experimental drugs, such as ITR (HY-17514, purity = 99.59%), posaconazole (POS, HY-17373, purity = 99.95%), were obtained from MedChemExpress (MCE), NJ, USA. VOR (V129745, purity≥98%), ISA (I337027, ≥98%), FLU (F129360, ≥98%), and UA (U275043, ≥98%) were purchased from Aladdin Biotech Co., Ltd., China. The working concentration ranges were 0.06 ~ 8 μg/mL for most drugs, with exceptions for POS (0.02 ~ 2 μg/mL) and UA (1 ~ 64 μg/mL). The solvents, dimethyl sulfoxide (DMSO, ≥99.9%) and MOPS buffer (pH 7.0), were obtained from Sinopharm Chemical Reagent Co., Ltd.

### *In vitro* susceptibility testing

Antifungal susceptibility was tested via the CLSI M38 broth microdilution method (22). Standardized fungal conidia ((3 ~ 5) × 10⁴ CFU/mL) were exposed to drug combinations in a checkerboard pattern within 96-well plates. After incubation at 35 °C (24 h for *Candida* spp. and *C. neoformans*, 48 h for *Aspergillus* spp., 72 h for *E. dermatitidis*), the Minimum Inhibitory Concentration (MIC) was determined, defined as the lowest drug concentration that inhibits ≥50% of fungal growth. Drug interactions were assessed using the Fractional Inhibitory Concentration Index (FICI) (Gómara et al., 2019), with interpretations as follows: FICI ≤ 0.5, synergy; 0.5 < FICI ≤ 4.0, no interaction; FICI > 4.0, antagonism.

### Detection of ROS content

Following a 48 h cultivation period on SAB medium at 35 °C, *A. fumigatus* conidia were harvested and subsequently diluted in 1640 liquid medium to 5 × 10⁴ cfu/mL. The suspensions were then subjected to a 16 h incubation at 37 °C, and drug treatments were applied as needed. After incubation, the fluorescent probe 2,7-dichlorodihydrofluorescein diacetate (obtained from Yeasen Biotechnology, Shanghai, China) was added to each sample, followed by an additional 1 h incubation at 37 °C. Fluorescence signals were captured on a Beckman Cytomics FC 500 BD FACSCanto II at an excitation/emission configuration of 488 nm and 525 nm, respectively.

### Rhodamine 6G efflux assay

The procedure outlined by Kolaczkowski et al. (1996) was adopted, with modifications, Briefly, conidia harvested from SAB plates at OD_600_ 0.5 were pelleted via centrifugation and subsequently rinsed three times with distilled water. Pellets were re-suspended in HEPES-NaOH (50 mM, pH 7.0) containing 5 mM 2-DG and 10 μM R6G (1.0 × 10^7^ cells/mL), shaken at 30 °C for 90 min, washed twice, and 400 μL aliquots were pre-incubated (30 °C, 5 min) before efflux initiation with 2 mM glucose. At set intervals after glucose addition, cells were pelleted by brief centrifugation, and 100 μL supernatant aliquots (in triplicate) were transferred to 96-well flat-bottom plates (BKMAM, Hunan, China). Fluorescence of extruded R6G was quantified on a microplate reader (ALLSHENG, Wuhan) with excitation at 529 nm.

### Statistical analysis

Statistical analyses were performed using GraphPad Prism 8.0 for descriptive statistics, graphing, and comparisons of continuous variables. Continuous data are presented as mean ± SD and were compared using Student’s *t*-test. Interaction outcomes were classified as synergy (S, FICI ≤ 0.5), indifference (I, 0.5 < FICI ≤ 4.0), and antagonism (A, FICI > 4.0). Interaction rates are presented as n/N (%) with two-sided 95% Wilson score confidence intervals. Overall comparisons of synergy and antagonism rates among drug combinations were performed using Fisher–Freeman–Halton exact tests, followed by two-sided Fisher exact tests for pairwise comparisons with Benjamini–Hochberg correction. Species-group comparisons were performed using the same exact-test framework. A two-sided *p* < 0.05 was considered statistically significant; *p < 0.05, **p < 0.01, ***p < 0.001; ns, not significant.

## Results

### Interaction between UA and azole drugs against *Aspergillus* spp

The MICs of UA and azole drugs against 66 clinical *Aspergillus* isolates were determined *in vitro* (Supplementary Table S1). UA alone exhibited no appreciable antifungal activity. The MIC ranges for the azole drugs alone were 0.5 ~ 8 μg/mL for ITR, 0.125 ~ 8 μg/mL for VOR, 0.125 ~ 2 μg/mL for POS, and 0.13 ~ 8 μg/mL for ISA (Supplementary Table S1). In combination, UA demonstrated synergistic effects with ITR and POS, with synergy rates of 9.38% (6/64) and 70.31% (45/64), respectively (Figures 1A–C). In contrast, no synergy was observed for UA combined with VOR or ISA; instead, these combinations exhibited certain degrees of antagonism, with antagonism rates of 9.38% (6/64) and 6.25% (4/64), respectively (Figures 1A,D,E). Specifically, synergy between UA and ITR was observed against *A. fumigatus* (14.29%, 4/28) and *A. flavus* (13.33%, 2/15), but not against *A. niger* or *A. terreus* (Figure 1B). Conversely, UA and POS combination showed synergistic effects against *A. fumigatus*, *A. flavus*, and *A. terreus*, with synergy rates of 82.14% (23/28), 66.67% (10/15), and 92.31% (12/13), respectively (Figure 1C). Importantly, when synergy occurred, the MICs of ITR and POS were reduced to 0.125 ~ 0.5 μg/mL and 0.03 ~ 0.5 μg/mL, respectively, significantly lowering the effective concentrations of the azole drugs (Supplementary Table S1). These findings suggest that UA can enhance the antifungal activity of azoles, particularly ITR and POS, against *Aspergillus* spp.

**Figure 1:**
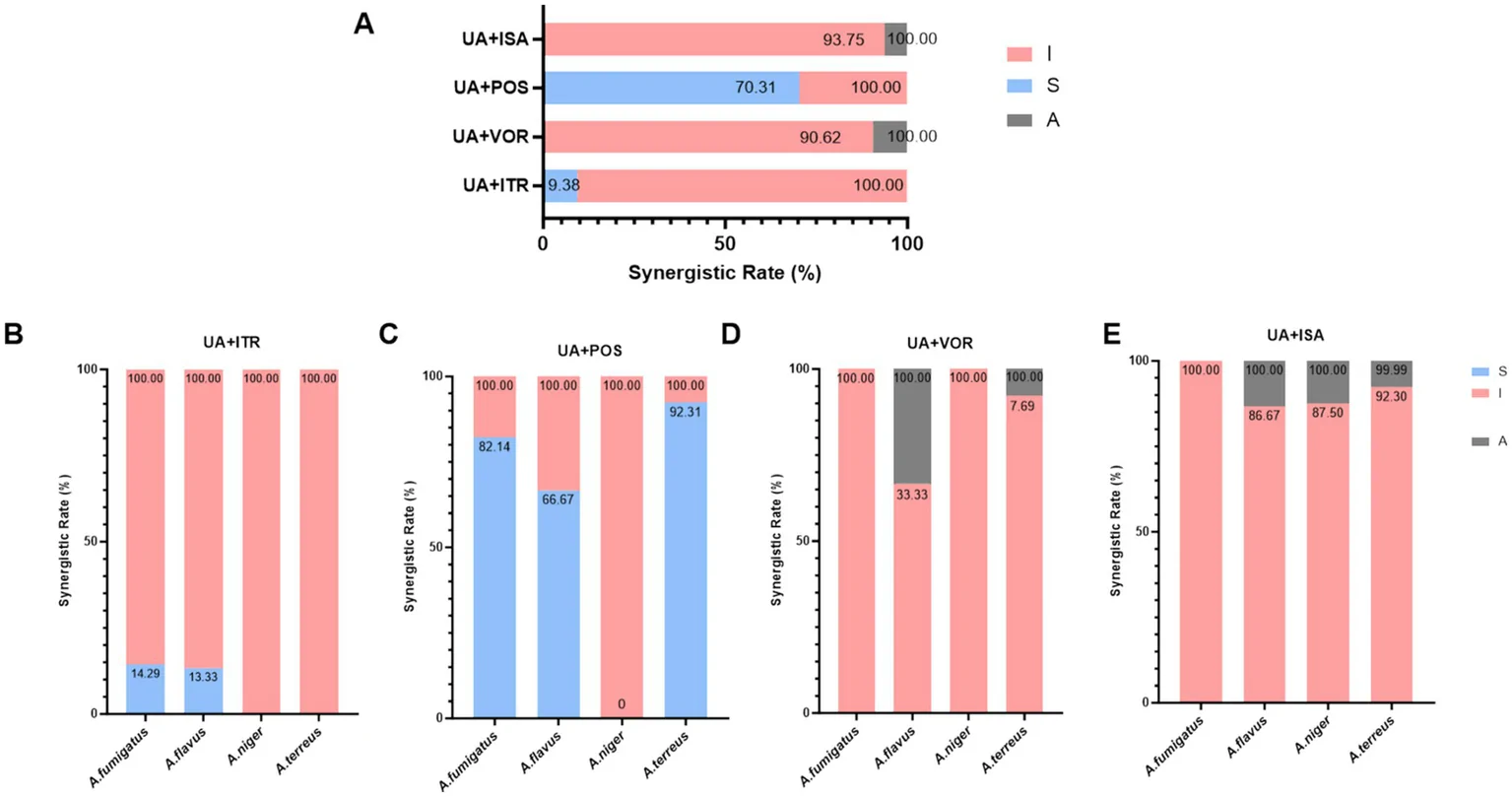
Synergy rates of UA and azoles against *Aspergillus* spp. **(A)** Overall synergy rates for UA-azole combinations. **(B–E)** Breakdown of synergy rates for UA with ITR **(B)**, VOR **(C)**, POS **(D)** and ISA **(E)** against four major *Aspergillus* species (*A. flavus*, *A. terreus*, *A. niger*, *A. fumigatus*). Interaction categories: S (Synergy, FICI ≤ 0.5); I (Indifference, FICI > 0.5 and ≤4); A (antagonism, FICI > 4.0).

### Interaction between UA and azole drugs against *Candida* spp

In the *in vitro* evaluation of UA and azole drugs against *Candida* spp., UA alone showed limited activity, with MICs ≥32 μg/mL against most isolates (23/24, 95.8%). The MIC ranges for the azoles alone were as follows: ITR 0.06 ~ 4 μg/mL, VOR 0.03 ~ 8 μg/mL, POS 0.03 ~ 0.5 μg/mL, and FLU 0.25 ~ 8 μg/mL (Supplementary Table S2). When used in combination, UA exhibited synergistic effects with all tested azoles. The synergy rates were 16.67% (4/24) for UA/ITR, 25% (6/24) for UA/VOR, 66.67% (16/24) for UA/POS, and 14.28% (2/14) for UA/FLU (Figure 2A). Specifically, UA significantly enhanced the antifungal activity of ITR against *C. albicans* (16.67%, 1/6) and *C. auris* (30%, 3/10) (Figure 2B). A similar enhancement was observed for UA/VOR; however, this combination exhibited antagonism against *C. tropicalis* (66.67%, 2/3) and *C. auris* (10%, 1/10) (Figure 2C). Notably, the UA/POS combination demonstrated strong synergy, with rates of 83.33% (5/6) against *C. albicans*, 60% (3/5) against *C. glabrata*, and 80% (8/10) against *C. auris*. No antagonism was observed for UA/POS against any of the tested *Candida* spp. (Figure 2D). In addition, UA/FLU showed synergy against *C. albicans* at a rate of 33.33% (2/6) (Figure 2E). Importantly, in cases where synergy occurred, the presence of UA significantly reduced the MICs of the azole drugs to the following ranges: 0.06 ~ 1 μg/mL for ITR, 0.06 ~ 2 μg/mL for VOR, 0.03 ~ 0.13 μg/mL for POS, and 0.25 ~ 0.5 μg/mL for FLU (Supplementary Table S2).

**Figure 2:**
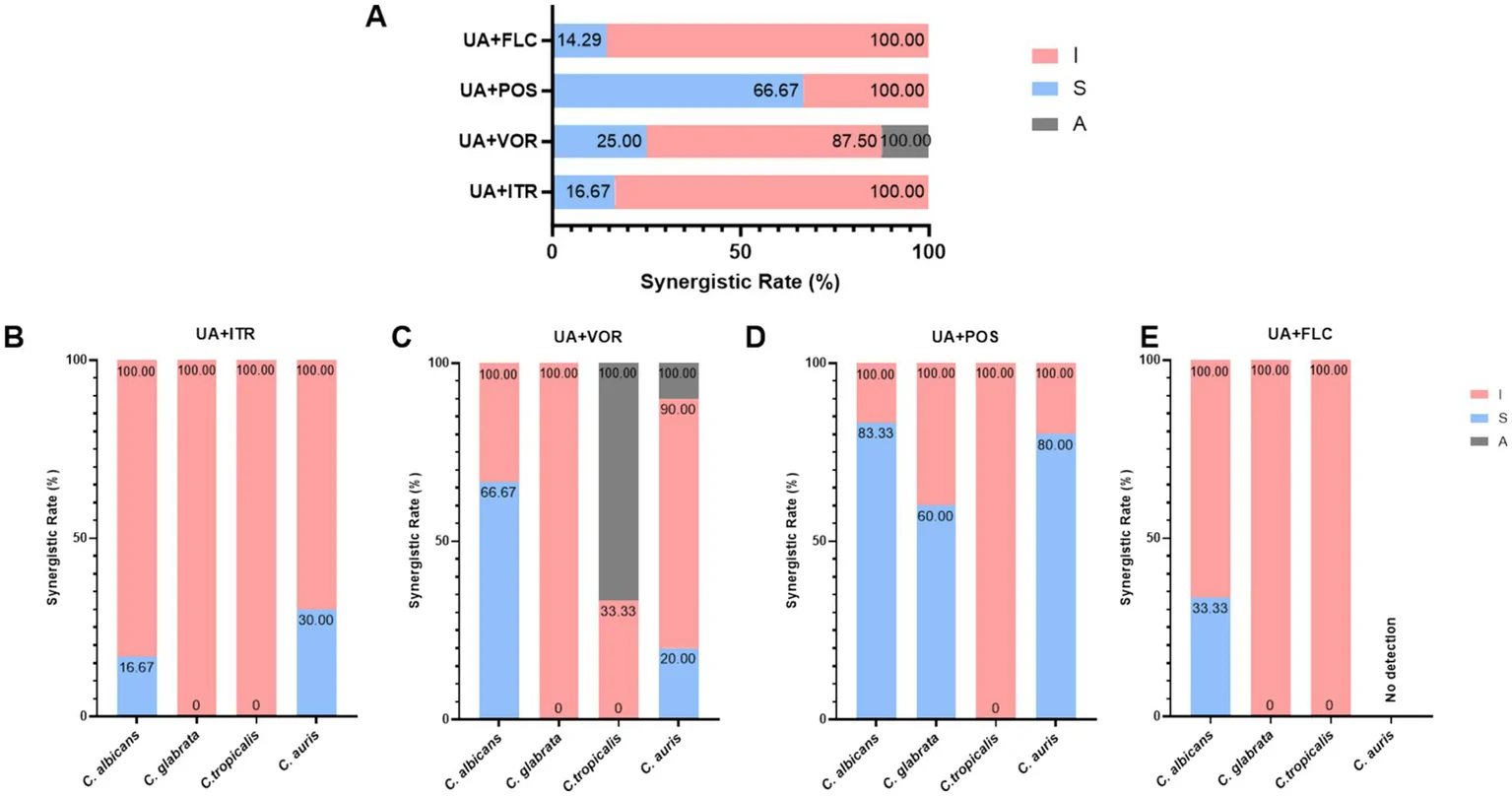
Synergy rates of UA and azoles against *Candida* spp. **(A)** Overall synergy rates for UA-azole combinations. **(B–E)** Breakdown of synergy rates for UA with ITR **(B)**, VOR **(C)**, POS **(D)** and FLU **(E)** against four major *Candida* spp. (*C. albicans*, *C. glabrata*, *C. tropicali* and *C. auris*). Interaction categories: S (Synergy, FICI ≤ 0.5); I (Indifference, FICI > 0.5 and ≤4); A (antagonism, FICI > 4.0).

### Interaction between UA and azole drugs against *E. dermatitidis*

*In vitro* susceptibility testing against *E. dermatitidis* revealed no antifungal activity for UA alone. The MIC ranges for the four azole drugs alone were 0.5 ~ 2 μg/mL for ITR, 0.13 ~ 0.5 μg/mL for VOR, 0.125 ~ 0.5 μg/mL for POS, and 0.13 ~ 2 μg/mL for ISA (Supplementary Table S3). In combination, UA/ITR and UA/POS showed synergy rates of 21.43% (3/14) and 92.86% (13/14), respectively. In contrast, no synergistic effects were observed for UA combined with VOR or ISA (Figure 3). Importantly, UA reduced the MICs of ITR and POS to 0.13 ~ 0.25 μg/mL and 0.03 ~ 0.13 μg/mL, respectively (Supplementary Table S3).

**Figure 3:**
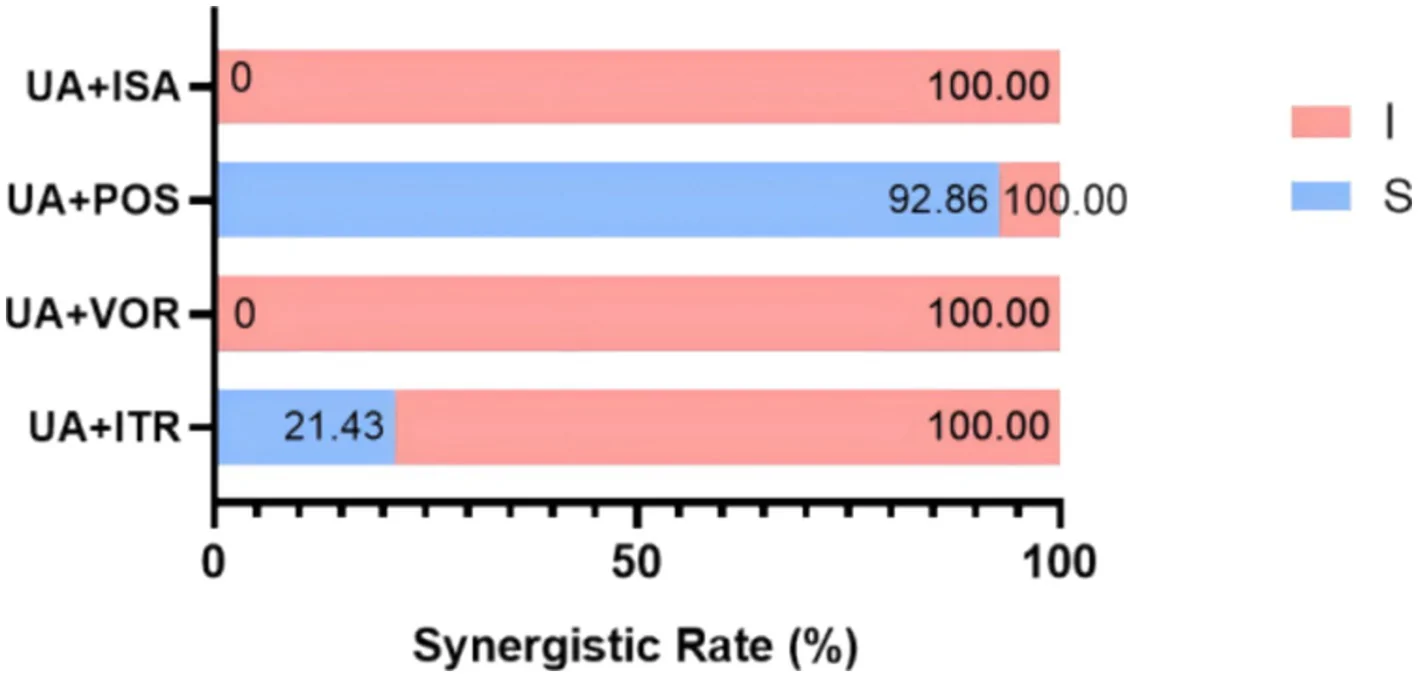
Synergy rates of UA and azoles against *E. dermatitidis*. The synergy rates for UA interacting with ITR, VOR, POS and ISA against *E. dermatitidis*. Interaction categories: S (Synergy, FICI ≤ 0.5); I (Indifference, FICI > 0.5 and ≤4).

### Interaction between UA and azole drugs against *C. neoformans*

The MICs of UA and azole drugs were also determined *in vitro* against *C. neoformans*. UA alone exhibited no inhibitory activity. The MIC ranges for the azoles alone were 1 ~ 4 μg/mL for ITR, 0.06 μg/mL for VOR, 0.25 ~ 0.5 μg/mL for POS, and 1 ~ 2 μg/mL for FLU (Supplementary Table S4). Notably, no synergistic effects were observed for UA in combination with any of the tested azoles. Conversely, significant antagonism was detected, with UA/VOR and UA/FLU exhibiting antagonism rates as high as 100% (8/8) and 87.5% (7/8), respectively (Figure 4).

**Figure 4:**
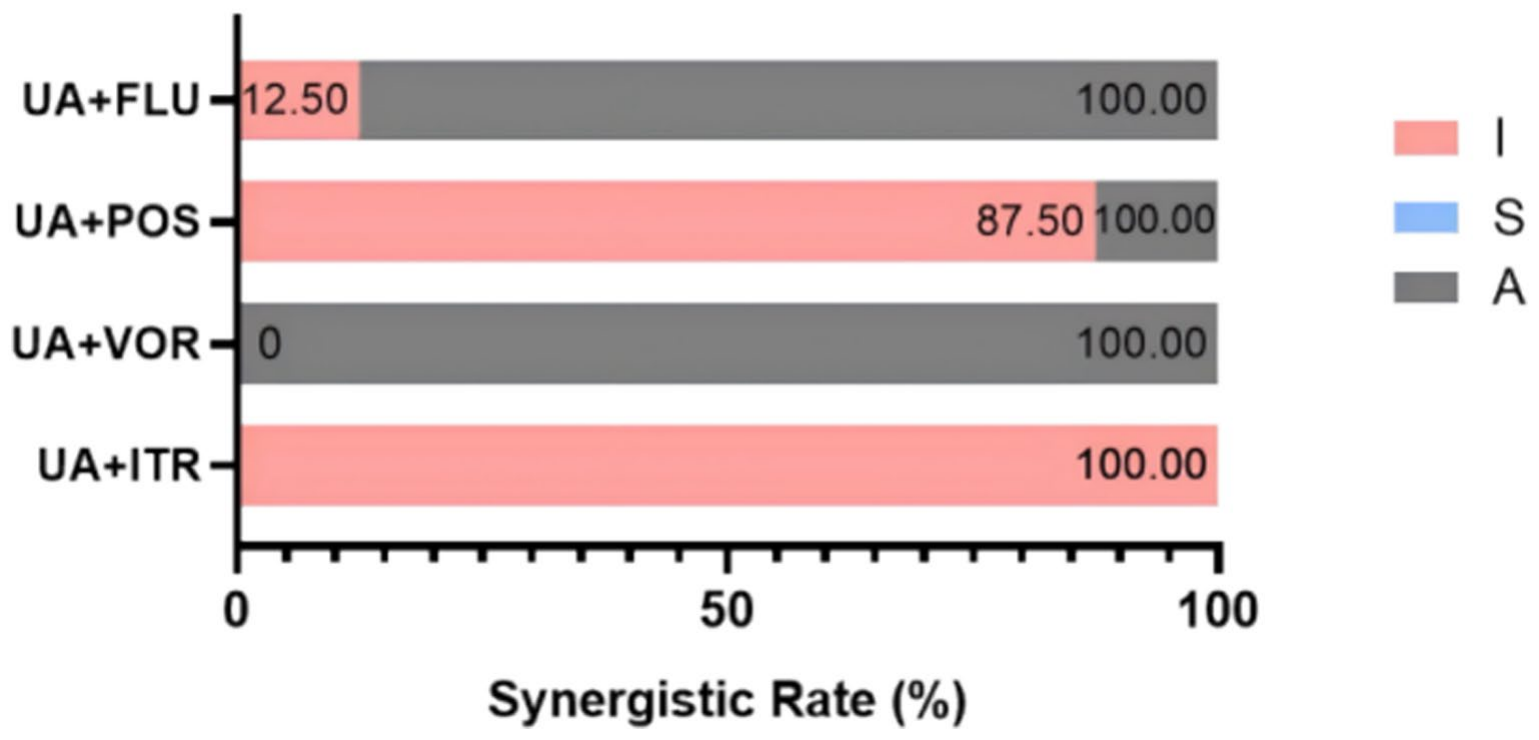
Synergy rates of UA and azoles against *C. neoformans*. The synergy rates for UA interacting with ITR, VOR, POS and ISA against *C. neoformans*. Interaction categories: S (Synergy, FICI ≤ 0.5); I (Indifference, FICI > 0.5 and ≤4), A (antagonism, FICI > 4.0).

### Summary of interactions between UA and azoles against pathogenic fungi

An integrated analysis of the *in vitro* susceptibility results across all clinical isolates revealed that the combinations of UA/ITR and UA/POS exhibited synergistic effects against *Aspergillus* spp., with synergy rates of 9.38% (6/64) and 70.31% (45/64), respectively, and no observed antagonism. For *Candida* spp., the synergy rates for UA/ITR, UA/VOR, and UA/POS were 16.67% (4/24), 25% (6/24), and 66.67% (16/24), respectively (Figure 5). However, the UA/VOR combination resulted in an antagonism rate of 12.5% (3/24) (Figure 5). For *E. dermatitidis*, UA/ITR and UA/POS demonstrated synergy rates of 21.43% (3/14) and 92.86% (13/14), respectively, with no antagonism detected for either combination (Figure 5). In contrast, no synergy was observed for any UA-azole combination against *C. neoformans*. Instead, significant antagonism was noted, with UA/VOR and UA/FLU exhibiting particularly high antagonism rates of 100% (8/8) and 87.5% (7/8), respectively (Supplementary Table S5 and Figure 5).

**Figure 5:**
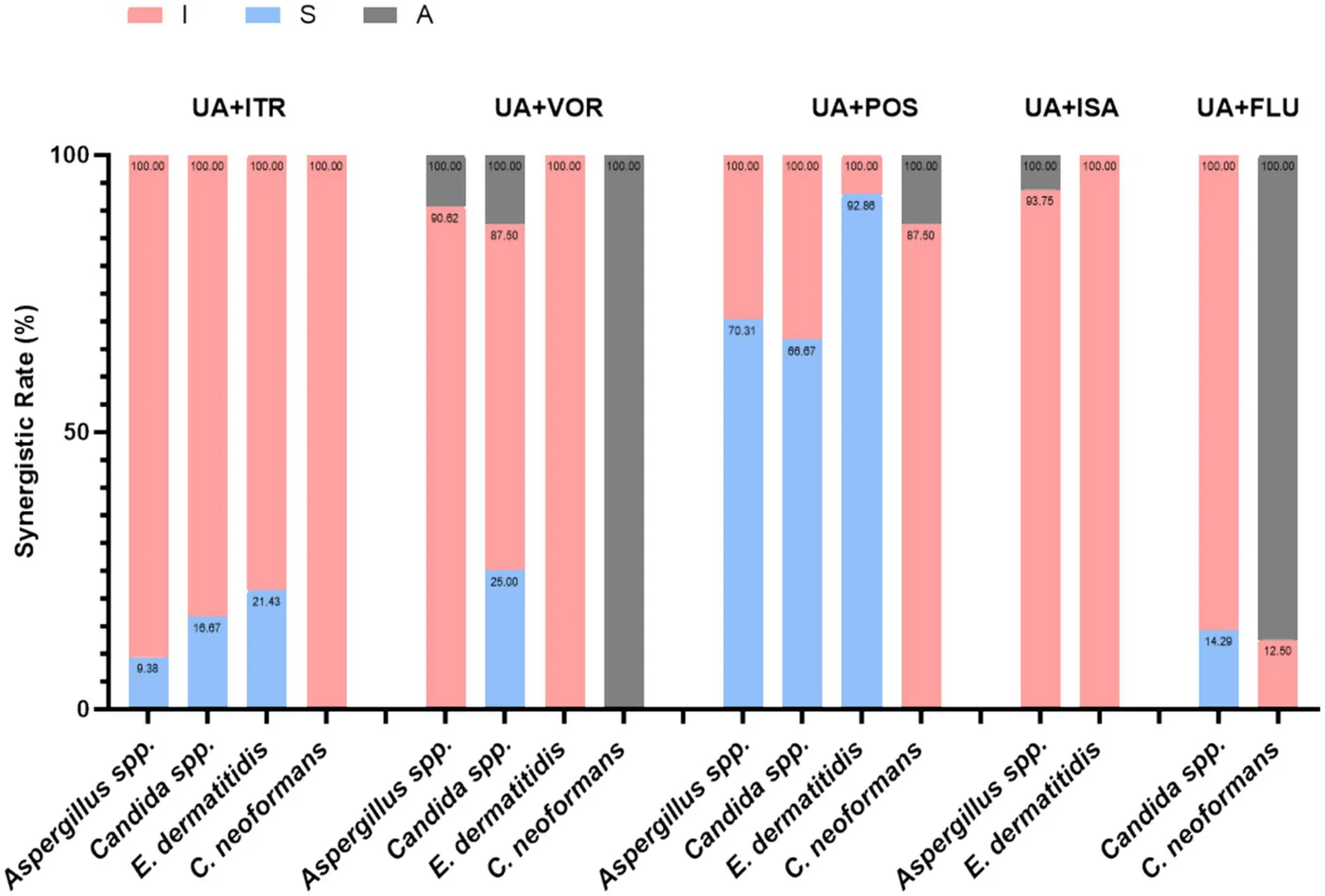
Summary of the Species-Specific Interactions Between UA and Azole Drugs Against Pathogenic Fungi. Interaction categories: S (Synergy, FICI ≤ 0.5); I (Indifference, FICI > 0.5 and ≤ 4), A (antagonism, FICI > 4.0).

Overall, the synergy rates for UA/ITR, UA/VOR, UA/POS, UA/ISA and UA/FLU across all available isolates were 11.82% (13/110; 95% CI 7.04 ~ 19.18%), 5.45% (6/110; 95% CI 2.52 ~ 11.39%), 67.27% (74/110; 95% CI 58.05 ~ 75.33%), 0.00% (0/78; 95% CI 0.00 ~ 4.62%) and 9.09% (2/22; 95% CI 2.53 ~ 27.81%), respectively (Supplementary Table S5). Synergy differed significantly among the five combinations (Fisher–Freeman–Halton exact test, *p* < 0.001), and UA/POS had a significantly higher synergy rate than each other combination after Benjamini–Hochberg correction (adjusted *p* < 0.001 for all four comparisons involving UA/POS). Antagonism also differed significantly among combinations (*p* < 0.001) (Supplementary Table S6). Species-group exact tests showed significant heterogeneity for UA/POS synergy, UA/VOR synergy and antagonism, and UA/FLU antagonism (all *p* < 0.001; Supplementary Table S7). Detailed rates, confidence intervals, and pairwise comparisons are provided in Supplementary Tables S5–S7, Table 1, and Figure 6.

**Table 1:** Summary of interactions between UA and azoles against pathogenic fungi.

Strains	ITR (%)			VOR (%)			POS (%)			ISA (%)			FLU (%)		
	I	S	A	I	S	A	I	S	A	I	S	A	I	S	A
*Aspergillus* spp. (*n* = 64)	90.62	9.38	0	89.06	0	10.94	29.69	70.31	0	93.75	0.00	6.25	NA	NA	NA
*Candida* spp. (*n* = 24)	83.33	16.67	0	62.5	25	12.5	33.33	66.67	0	NA	NA	NA	85.71	14.29	0.00
*E. dermatitidis* (*n* = 14)	78.57	21.43	0	100	0	0	7.14	92.86	0	100.00	0.00	0.00	NA	NA	NA
*C. neoformans* (*n* = 8)	100	0	0	0	0	100	87.5	0	12.5	NA	NA	NA	12.50	0.00	87.50

NA indicates that no tests were conducted for the corresponding strain. FLU was tested against Candida spp. (*n* = 14) and *C. neoformans* (*n* = 8). ISA was tested against molds (*Aspergillus* spp. and *E. dermatitidis*). The UA+FLU combination was therefore analyzed with *n* = 14 for *Candida* spp. Two-sided 95% Wilson confidence intervals and exact between-group comparisons are provided in Supplementary Tables S5–S7.

**Figure 6:**
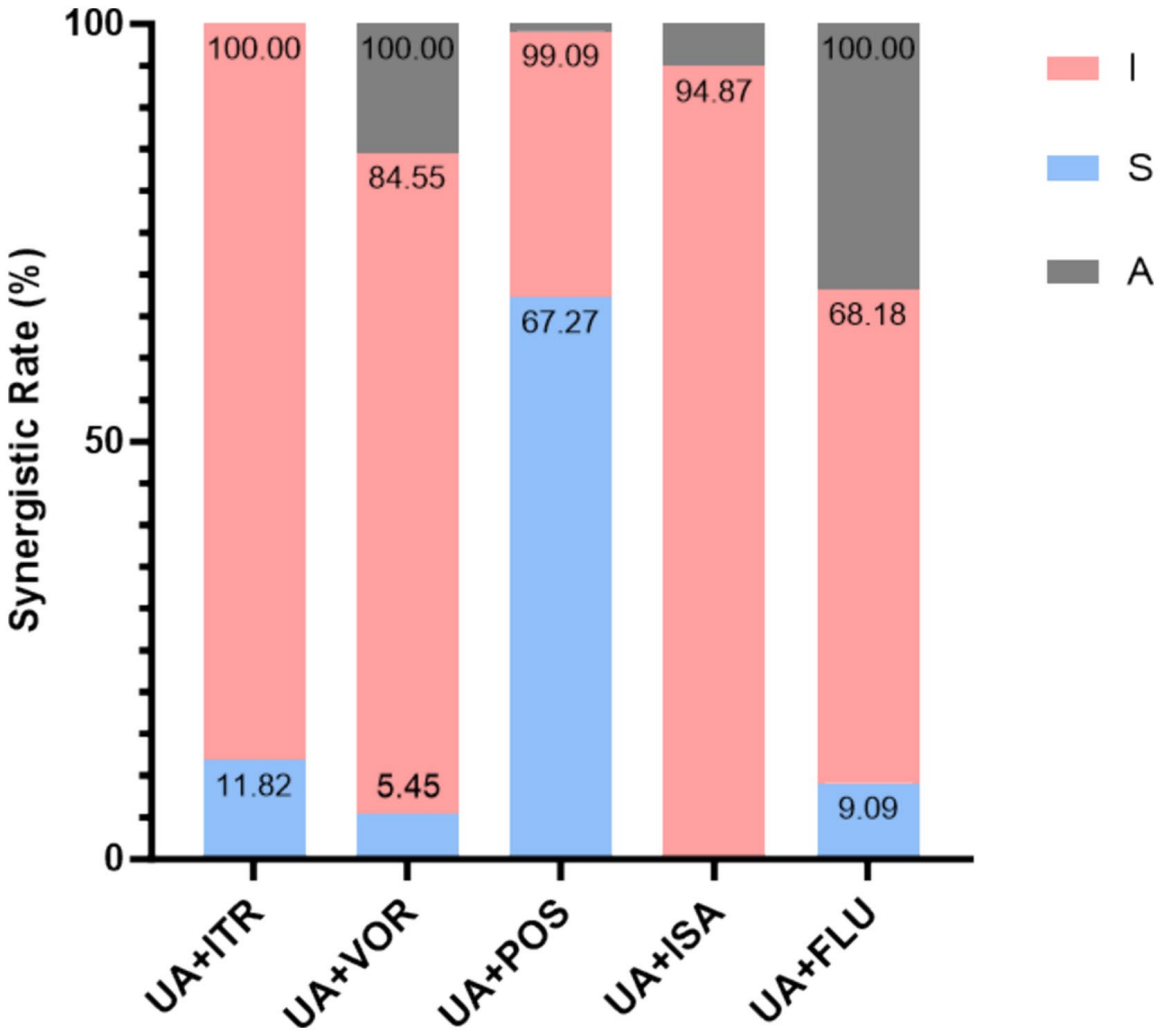
Interaction Profiles of UA with Different Azoles. Interaction categories: S (Synergy, FICI ≤ 0.5); I (Indifference, FICI > 0.5 and ≤4), A (antagonism, FICI > 4.0).

### Synergistic effects of UA + POS against sensitive and resistant strains

Based on the clinical susceptibility breakpoints, all fungal isolates were stratified into POS-sensitive and POS-resistant groups according to their POS MICs. Strains with POS MIC ≤ 0.25 μg/mL were defined as sensitive, while those with MIC > 0.25 μg/mL were classified as resistant. The summarized results are presented in Table 2. Overall, UA + POS exhibited markedly higher synergy rates among POS-sensitive strains than among POS-resistant strains across most tested species. Among the 62 POS-sensitive isolates, 43 (69.35%) demonstrated synergistic effects, whereas among the 48 POS-resistant isolates, only 26 (54.17%) showed synergy (Table 2).

**Table 2:** Summary of the synergistic effects of UA combined with POS on sensitive strains and drug-resistant strains.

Strains	Sensitive strains (the MIC of POS ≤ 0.25)				Resistant strains (the MIC of POS > 0.25)			
	Total	No. of synergistic strains	Synergy rate (%)	95% CI of synergy rate (%)	Total	No. of synergistic strains	Synergy rate (%)	95% CI of synergy rate (%)
*A. fumigatus*	22	22	100	84.56–100%	6	1	16.67	3.01–56.35%
*A. flavus*	4	4	100	39.76–100%	11	6	54.55	28.01–78.73%
*A. niger*	1	0	0	0.00–97.50%	7	0	0	0.00–40.96%
*A. terreus*	1	1	100	2.50–100.00%	12	11	91.67	64.61–98.51%
*C. albicans*	2	2	100	15.81–100.00%	4	3	75	30.06–95.44%
*C. glabrata*	5	3	60	23.07–88.24%	0	0	0	NA
*C. tropicalis*	3	0	0	0.00–70.76%	0	0	0	NA
*C. auris*	5	0	0	0.00–52.18%	5	3	60	23.07–88.24%
*E. dermatitidis*	12	11	91.67	64.61–98.51%	2	2	100	15.81–100.00%
*C. neoformans*	7	0	0	0.00–40.96%	1	0	0	0.00–97.50%

CI: 95% CIs are two-sided Wilson score intervals, except for rates of 0% or 100%, which use Clopper–Pearson exact intervals. Subgroups with *n* = 0 are reported as NA rather than as a 0% rate.

For POS-sensitive strains, UA + POS displayed potent synergistic activity against *A. fumigatus, A. flavus, A. terreus, C. albicans*, and *E. dermatitidis*, with synergy rates reaching 100, 100, 100, 100, and 91.67%, respectively. In contrast, low or no synergy was observed in *A. niger* (0%, *n* = 1), *C. tropicalis* (0%, *n* = 3), *C. auris* (0%, *n* = 5), and *C. neoformans* (0%, *n* = 7). *C. glabrata* showed a moderate synergy rate of 60% (3/5). For POS-resistant strains, the combination also demonstrated notably high synergy rates in *A. terreus* (91.67%, 11/12) and *E. dermatitidis* (100%, 2/2). *A. flavus* exhibited a moderate synergy rate of 54.55% (6/11), while *C. albicans* and *C. auris* showed synergy rates of 75% (3/4) and 60% (3/5), respectively. In contrast, *A. fumigatus* resistant isolates displayed a markedly lower synergy rate of only 16.67% (1/6), despite their sensitive counterparts showing a 100% synergy rate. No synergistic activity was observed in resistant isolates of *A. niger* (0/7) or *C. neoformans* (0/1). Notably, no resistant isolates of *C. glabrata* or *C. tropicalis* were available in this study (Table 2).

Collectively, these findings indicate that the synergistic interaction between POS and UA is species-dependent and modulated by the POS susceptibility profile of the clinical isolates. Specifically, strong synergy was observed in both sensitive and resistant backgrounds for *A. fumigatus, A. terreus, C. albicans*, and *E. dermatitidis*, whereas species such as *A. niger, C. tropicalis, C. auris*, and *C. neoformans* exhibited minimal or no synergy regardless of susceptibility status.

### UA + POS enhances intracellular ROS production

To investigate the mechanism underlying the potent synergy between POS and UA, we randomly selected 9 clinical isolates from the 110 strains collection for ROS accumulation assays, including 7 strains with high synergy and 2 non-synergistic strains. The selected isolates were: 293 (S), 382 (S), 469 (S), 121,889 (S), 130,110 (S), FL13 (S), PY1b (S), AN5 (I), and Y3 (I). POS treatment alone significantly increased intracellular ROS levels in all tested strains. Notably, compared with POS alone, the UA + POS combination further elevated ROS levels in 5 of the 7 synergistic strains (293, 382, 121,889, 130,110, and PY1b). In contrast, no additional increase was observed in the remaining two synergistic strains (469 and FL13) or in the two non-synergistic strains upon co-stimulation (Figure 7).

**Figure 7:**
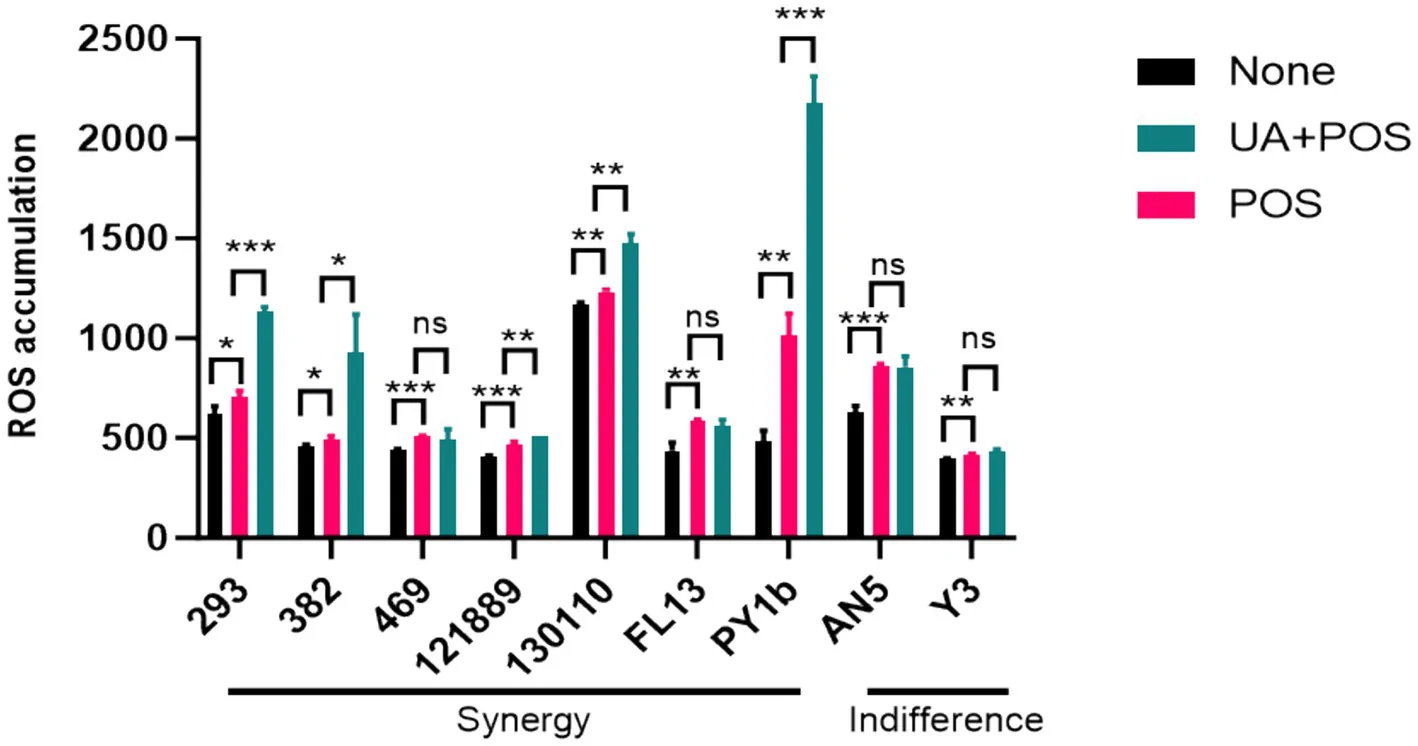
ROS levels after drug treatment. Cells were labeled with the fluorescent probe DCFH-DA, and fluorescence intensity was quantified by flow cytometry (excitation/emission: 488/525 nm). Data are presented as mean fluorescence intensity, which reflects the relative ROS abundance within the cell population. Significance was determined by *t*-test: *p < 0.05, **p < 0.01, ***p < 0.0001; ns, not significant.

### UA + POS suppresses efflux pump function

To assess the role of efflux pumps in the observed synergy, we measured efflux pump activity in the 9 selected clinical isolates. Our results demonstrated that, at 25 min post-treatment, compared with POS alone, the UA + POS combination significantly reduced efflux pump function in 4 of the synergistic strains (382, 469, FL13, and PY1b). In contrast, no functional alteration was observed in the remaining strains upon co-stimulation (Figure 8).

**Figure 8:**
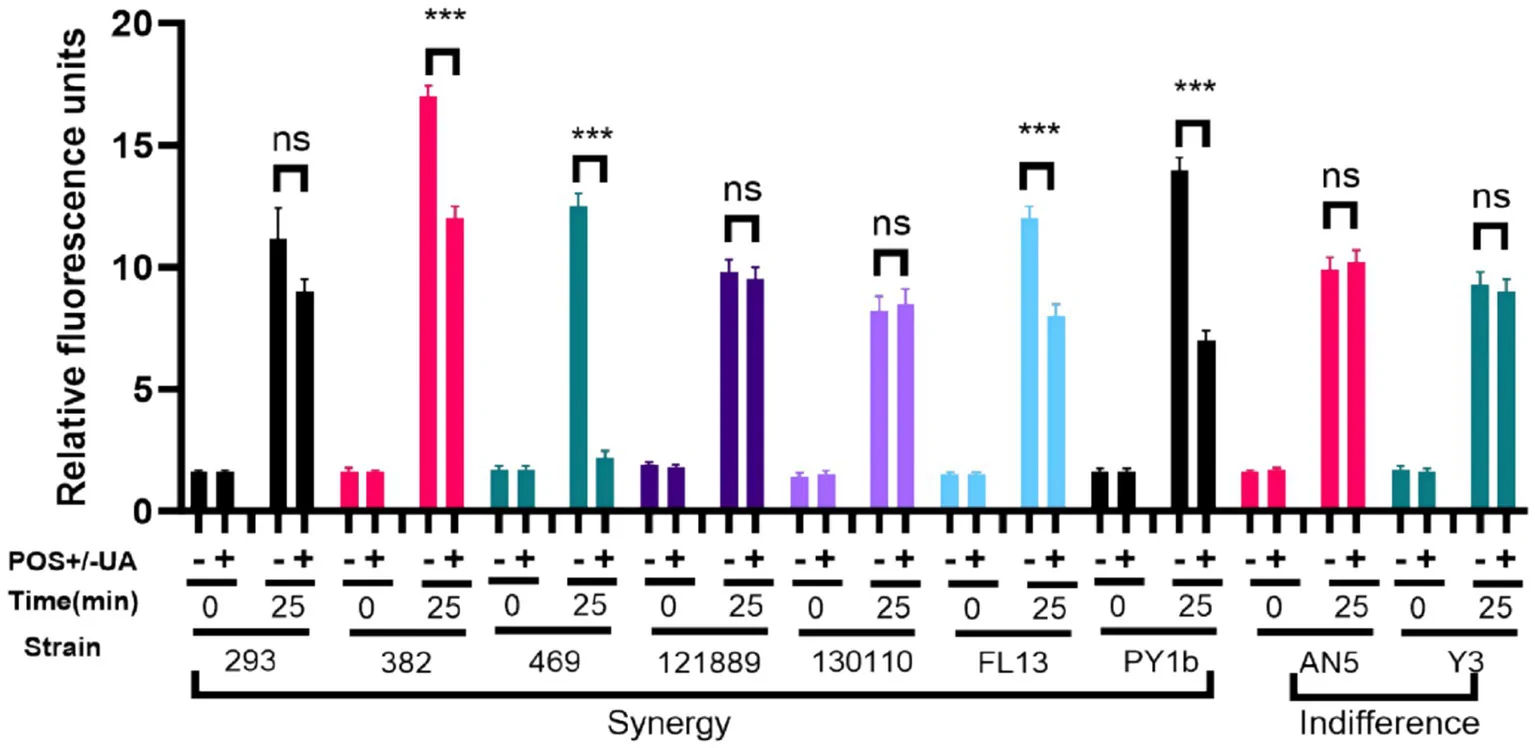
Influence of UA combined with POS on R6G exhaust. De-energized cells were preloaded with R6G, and efflux was initiated by adding 2 mM glucose. Fluorescence of extruded R6G in cell supernatants was measured at 0 and 25 min after glucose addition. Data are presented as mean ± SD (*n* = 3). Significance was determined by *t*-test: ***p < 0.0001; ns, not significant.

## Discussion

This study systematically demonstrates through *in vitro* susceptibility testing that the natural product usnic acid can function as an effective antimicrobial adjuvant. It exhibits synergistic effects with all tested azole drugs (ITR, VOR, POS, ISA and FLU), significantly enhancing their efficacy against clinical isolates of *Aspergillus* spp., *Candida* spp., *E. dermatitidis* and *C. neoformans*. A particularly notable finding was the broad and potent synergistic effect of the UA + POS combination, which demonstrated a remarkable inhibition rate of 92.86% against *A. terreus* and *E. dermatitidis* (Figures 1B, 3).

Further analysis revealed that the synergistic interaction is neither universally applicable across all species nor uniformly effective between sensitive and resistant strains. Broad-spectrum synergy was observed in *A. terreus* and *E. dermatitidis* regardless of susceptibility status, whereas in *A. fumigatus,* synergy was largely restricted to sensitive isolates. Minimal or no synergy was detected in *A. niger, C. tropicalis, C. auris* (sensitive isolates only), and *C. neoformans* (Table 2). These findings provide a rational basis for tailoring the UA + POS combination strategy to specific fungal species and resistance phenotypes, rather than adopting a one-size-fits-all approach. This stratified strategy holds promise for optimizing clinical outcomes while minimizing unnecessary drug exposure and potential adverse effects.

Beyond its clinical relevance, the potent synergy observed against *A. flavus* may have agricultural implications. As a major phytopathogen infecting maize, peanuts, and other crops, *A. flavus* causes aflatoxin contamination that results in substantial economic losses and food safety risks worldwide (Abdullaziz et al., 2026). Current agricultural control measures, including breeding of resistant varieties, optimization of cultivation management, and biological control using non-toxigenic strains, have achieved certain results but remain limited by environmental variability, high costs, or inconsistent efficacy. Our data demonstrate that the UA + POS combination achieved a 100% synergy rate in sensitive *A. flavus* isolates and 54.55% even in resistant ones. Although derived from *in vitro* susceptibility testing rather than field trials, these findings provide preliminary experimental evidence supporting the extension of efflux pump inhibitor–azole combination strategies to agricultural settings, warranting further validation in crop protection contexts.

At the mechanistic level, we preliminarily explored the molecular basis of the UA–POS synergy. Our results demonstrated that the UA + POS combination exerts its synergistic antifungal activity through enhancing intracellular ROS accumulation and suppressing efflux pump function. Regarding ROS accumulation, 71.43% (5/7) of the synergistic strains exhibited a significant elevation in intracellular ROS levels upon combined stimulation, even exceeding those observed with POS alone (Zhang et al., 2025), whereas no excessive ROS accumulation was detected in non-synergistic strains. It has been reported that usnic acid directly targets mitochondria by inhibiting respiratory chain complexes I and III (Qi et al., 2020), and also uncouples oxidative phosphorylation, thereby exacerbating oxidative stress (Han et al., 2004). This combined action might provoke an overwhelming oxidative burst that exceeds the fungal antioxidant capacity, leading to irreversible cellular damage and cell death.

Furthermore, Sen et al. (2024) recently demonstrated that overexpression of efflux pump genes (*mdr1* and *mfs*) is a key driver of azole resistance in environmental Black *Aspergillus* species. In line with this, our study revealed that 57.14% (4/7) of the synergistic strains (382, 469, FL13, and PY1b) exhibited significant efflux pump inhibition upon UA + POS co-stimulation, which is consistent with previous reports demonstrating that UA inhibits efflux pump systems in bacteria (Zhang et al., 2023; Bankan et al., 2021). In addition, UA itself is membrane-active and can disrupt the integrity of microbial cell membranes (Zhang et al., 2023; Cocchietto et al., 2002), likely increasing membrane permeability and facilitating the entry of azole drugs into fungal cells to reach their target (CYP51 enzyme), thereby effectively complementing the action of azoles.

However, the synergistic effect was not achieved through a unified mechanism across all strains. Three synergistic isolates (293, 121,889, and 130,110) showed no alteration in efflux pump function, however their intracellular ROS levels were significantly elevated upon UA + POS co-stimulation. This mechanistic diversity is highly consistent with the phenomenon that even different isolates carrying the same *cyp51A* mutations may exhibit distinct gene expression profiles (Sen et al., 2024). Importantly, two strains (382 and PY1b) simultaneously exhibited both enhanced ROS accumulation and efflux pump inhibition, suggesting that the mechanism of UA + POS synergy is complex and may involve crosstalk between these two pathways. Further investigations are warranted to elucidate the underlying regulatory networks at the molecular level.

The question of why UA exhibits the most profound synergy with POS is critical. This remarkable effect may be attributed to two synergistic factors. First, POS’s high lipophilicity promotes its integration into the fungal membrane (Shi et al., 2020), a process likely potentiated by UA’s membrane-perturbing activity, thereby enhancing POS uptake. Second, and perhaps more importantly, since POS is a primary substrate for many resistance-conferring transporters (Morris, 2009; Perez-Cantero et al., 2020), the proposed efflux pump inhibition by UA would disproportionately benefit POS, preventing its extrusion and leading to dramatic intracellular accumulation. This is clinically highly relevant, as enhancing the potency of POS, a first-line therapy for invasive mold infections, addresses a major unmet need in medical mycology. Specifically, although ITR and POS share the CYP51 target, their synergistic outcomes with UA differed markedly (11.82% vs. 67.27%), indicating that the shared target cannot account for the differential adjuvant effects of UA and that drug-specific cellular pharmacokinetics, particularly membrane partitioning and efflux-transporter substrate specificity, are likely decisive. POS is the most lipophilic azole and a well-documented substrate of fungal multidrug efflux transporters (Leung et al., 2015), so UA-mediated membrane perturbation and efflux inhibition would disproportionately enhance POS membrane partitioning and intracellular retention, whereas ITR exhibited high intrinsic MICs (0.5–8 μg/mL) in our isolates and the checkerboard/FICI format has limited resolution when the azole MIC is high, further narrowing the window for detecting ITR synergy (Langner et al., 2008). In contrast, VOR is the most hydrophilic azole tested and is thought to enter fungal cells largely by passive diffusion, so UA-induced membrane perturbation may reduce rather than increase its intracellular accumulation; in species with inducible efflux or robust stress responses, most prominently *C. neoformans*, co-exposure may also upregulate transporter expression or trigger stress-adaptation signaling that counteracts azole action, shifting the interaction toward antagonism (Felton et al., 2014). Indeed, UA + VOR antagonism was strongly species-restricted (8/8 *C. neoformans* isolates, 12.5% of *Candida* isolates, and none in *Aspergillus* or *E. dermatitidis*), which is consistent with the biology of *C. neoformans*. As a basidiomycete, its membrane lipid composition and large ABC/MFS transporter repertoire differ substantially from those of the ascomycete pathogens tested here, and efflux is a major determinant of azole susceptibility in this species (Cavalheiro et al., 2018; Melhem et al., 2024); we hypothesize that UA is recognized by or induces these efflux systems, or that its membrane effects trigger adaptive stress pathways, resulting in reduced net azole accumulation and the 100%/87.5% antagonism observed with VOR and FLU. Importantly, this *in vitro* finding carries direct clinical safety implications: the data argue strongly against combining UA with VOR or FLU in *cryptococcosis*, whereas UA + POS and UA + ITR showed no such antagonism against *C. neoformans* (0% antagonism for both), further supporting species-tailored rather than universal application of UA–azole combinations.

A key strength of this work is its demonstration that usnic acid’s adjuvant effect is not restricted to a single species but shows broad-spectrum potential against major fungal pathogens, a critical attribute for a universal anti-IFI strategy. The translational potential of this approach is twofold: it could enable lower, safer dosing of azoles while potentially overcoming resistance in formidable pathogens such as multidrug-resistant *E. dermatitidis* and *C. auris*.

We acknowledge that our study has limitations. The evidence remains at the *in vitro* stage, and the clinical applicability must be confirmed in animal models. More importantly, definitive proof for our mechanistic hypotheses, such as efflux pump inhibition leading to increased intracellular drug accumulation, awaits direct experimental validation in future work.

Therefore, we conclude that usnic acid represents a promising broad-spectrum antifungal adjuvant, and the combination therapy with POS merits prioritized investigation. Moving forward, it is imperative to define the underlying molecular mechanisms and to substantiate the therapeutic profile in animal models, with the clear objective of translating this natural product into a novel combination strategy to mitigate the escalating crisis of antifungal resistance.

## Data Availability

The original contributions presented in the study are included in the article/Supplementary material, further inquiries can be directed to the corresponding author.
