# Supplementary material for: Enhanced antifungal efficacy of azole drugs through combination with usnic acid
Source: Front Microbiol. 2026 Oct 19;17:1864975. doi: 10.3389/fmicb.2026.1864975 (PMC13724356; doi:10.3389/fmicb.2026.1864975)
Supplement: Supplementary file 1 [file Table_1.docx]

Table S1 Interaction between UA and azole drugs against Aspergillus spp.

| **Strains^a^** | **MIC^b^ alone (μg/mL)** | | | | |  | **MIC combinations (μg/mL) (FICI^c^)** | | | |
| --- | --- | --- | --- | --- | --- | --- | --- | --- | --- | --- |
|  | **UA** | **ITR** | **VOR** | **POS** | **ISA** |  | **UA/ITR** | **UA/VOR** | **UA/POS** | **UA/ISA** |
| *A. fumigatus* | | | | | | | | | | |
| 293 | >64 | 2 | 0.25 | 0.25 | 0.25 |  | 64/1 (I) | 64/0.25 (I) | 8/0.03 (S) | 32/0.25 (I) |
| AF0 | >64 | 2 | 0.25 | 0.125 | 0.5 |  | 32/1 (I) | 32/0.25 (I) | 32/0.03 (S) | 32/0.5 (I) |
| AF2 | >64 | 0.5 | 0.125 | 0.25 | 0.5 |  | 32/0.125 (S) | 1/0.125 (I) | 4/0.06 (S) | 32/0.5 (I) |
| AF3 | >64 | 0.5 | 0.25 | 0.125 | 0.5 |  | 32/0.25 (I) | 4/0.25 (I) | 32/0.03125 (S) | 32/0.5 (I) |
| AF4 | >64 | 0.5 | 0.125 | 0.125 | 0.5 |  | 32/0.125 (S) | 1/0.125 (I) | 8/0.03125 (S) | 32/0.5 (I) |
| AF5 | >64 | 0.5 | 0.25 | 0.25 | 0.5 |  | 32/0.25 (I) | 8/0.25 (I) | 16/0.06 (S) | 64/1 (I) |
| AF6 | >64 | 0.5 | 0.25 | 0.25 | 0.5 |  | 32/0.25 (I) | 1/0.25 (I) | 16/0.0625 (S) | 32/0.5 (I) |
| AF7 | >64 | 0.5 | 0.25 | 0.125 | 1 |  | 32/0.25 (I) | 1/0.25 (I) | 32/0.03125 (S) | 32/1 (I) |
| AF8 | >64 | 2 | 2 | 0.5 | 4 |  | 32/4 (I) | 1/2 (I) | 2/0.5 (I) | 32/1 (I) |
| TR34 | >64 | >8 | >8 | 1 | >8 |  | 16/128 (I) | 16/128 (I) | 32/0.5 (I) | 16/128 (I) |
| TR46 | >64 | >8 | 8 | 1 | >8 |  | 16/128 (I) | 32/8 (I) | 32/0.5 (I) | 16/128 (I) |
| TR34 | >64 | 8 | 2 | 1 | 2 |  | 64/4 (I) | 32/2 (I) | 64/0.25 (I) | 32/2 (I) |
| TR46 | >64 | 4 | >8 | 1 | >8 |  | 32/4 (I) | 16/128 (I) | 16/0.25 (S) | 16/128 (I) |
| AF16 | >64 | 1 | 0.25 | 0.25 | 0.5 |  | 64/1 (I) | 32/0.25 (I) | 8/0.06 (S) | 32/0.5 (I) |
| AF17 | >64 | 1 | 0.25 | 0.25 | 0.5 |  | 16/0.5 (I) | 32/0.25 (I) | 8/0.03 (S) | 32/0.5 (I) |
| AF18 | >64 | 2 | 2 | 0.25 | 2 |  | 32/4 (I) | 16/2 (I) | 8/0.06 (S) | 32/2 (I) |
| AF19 | >64 | 0.5 | 0.25 | 0.25 | 0.5 |  | 32/0.5 (I) | 32/0.25 (I) | 16/0.03 (S) | 32/0.5 (I) |
| AF20 | >64 | 0.5 | 0.25 | 0.125 | 0.5 |  | 32/0.5 (I) | 64/0.5 (I) | 16/0.03 (S) | 32/0.5 (I) |
| AF21 | >64 | 1 | 1 | 0.25 | 0.5 |  | 8/1 (I) | 32/1 (I) | 16/0.06 (S) | 32/0.5 (I) |
| AF22 | >64 | 1 | 0.5 | 0.25 | 1 |  | 32/1 (I) | 32/0.5 (I) | 16/0.06 (S) | 32/1 (I) |
| AF24 | >64 | 2 | 0.25 | 0.25 | 0.25 |  | 32/2 (I) | 4/0.5 (I) | 16/0.03 (S) | 4/0.5 (I) |
| AF25 | >64 | 1 | 1 | 0.25 | 1 |  | 64/1 (I) | 32/1 (I) | 16/0.03 (S) | 32/1 (I) |
| AF26 | >64 | 2 | 0.25 | 0.125 | 0.25 |  | 32/1 (I) | 64/0.13 (I) | 16/0.03 (S) | 64/0.13 (I) |
| AF27 | >64 | 1 | 0.5 | 0.25 | 1 |  | 4/1 (I) | 32/0.5 (I) | 16/0.06 (S) | 32/1 (I) |
| AF28 | >64 | 0.5 | 0.25 | 0.125 | 0.5 |  | 16/0.25 (I) | 32/0.25 (I) | 16/0.03 (S) | 32/0.5 (I) |
| AF29 | >64 | 1 | 1 | 2 | 1 |  | 16/0.25 (S) | 32/0.5 (I) | 32/2 (I) | 32/1 (I) |
| AF30 | >64 | 2 | 0.125 | 0.25 | 0.0625 |  | 4/2 (I) | 32/0.13 (I) | 2/0.06 (S) | 32/0.13 (I) |
| AF31 | >64 | 1 | 0.25 | 0.25 | 0.5 |  | 16/0.25 (S) | 32/0.25 (I) | 8/0.06 (S) | 32/0.5 (I) |
| *A. flavus* | | | | | | | | | | |
| FL2 | >64 | 2 | 1 | 0.25 | 0.5 |  | 32/2 (I) | 64/4 (A) | 16/0.03 (S) | 16/1 (I) |
| FL8 | >64 | 1 | 2 | 1 | 2 |  | 32/0.5 (I) | 32/2 (I) | 32/0.25 (S) | 32/2 (I) |
| FL9 | >64 | 1 | 2 | 2 | 2 |  | 8/1 (I) | 32/2 (I) | 32/1 (I) | 32/2 (I) |
| FL10 | >64 | 1 | 4 | 2 | 2 |  | 8/0.5 (I) | 32/4 (I) | 32/0.5 (S) | 32/2 (I) |
| FL11 | >64 | 4 | 0.5 | 0.5 | 0.25 |  | 32/4 (I) | 16/1 (I) | 16/0.13 (S) | 32/0.5 (I) |
| FL12 | >64 | 4 | 0.5 | 0.5 | 0.25 |  | 16/2 (I) | 64/2 (A) | 8/0.13 (S) | 32/0.25 (I) |
| FL13 | >64 | 4 | 0.25 | 0.5 | 0.25 |  | 8/2 (I) | 64/1 (A) | 4/0.13 (S) | 32/0.5 (I) |
| FL14 | >64 | 2 | 2 | 0.5 | 1 |  | 16/0.5 (S) | 32/2 (I) | 4/0.25 (I) | 32/1 (I) |
| FL15 | >64 | 4 | 0.5 | 0.5 | 0.25 |  | 32/4 (I) | 32/2 (A) | 16/0.13 (S) | 64/1 (A) |
| FL16 | >64 | 4 | 0.5 | 0.5 | 1 |  | 32/4 (I) | 64/2 (A) | 32/0.13 (S) | 32/1 (I) |
| FL17 | >64 | 8 | 0.5 | 2 | 2 |  | 32/8 (I) | 4/1 (I) | 64/1 (I) | 2/4 (I) |
| FL22 | >64 | 8 | 1 | 2 | 1 |  | 2/4 (I) | 32/1 (I) | 64/1 (I) | 32/1 (I) |
| FL23 | >64 | 2 | 1 | 1 | 0.5 |  | 32/2 (I) | 32/2 (I) | 32/0.5 (I) | 32/2 (A) |
| C15 | >64 | 0.5 | 0.5 | 0.25 | 2 |  | 8/0.25 (I) | 2/0.5 (I) | 32/0.06 (S) | 32/2 (I) |
| C16 | >64 | 0.5 | 0.5 | 0.25 | 0.5 |  | 32/0.13 (S) | 1/0.5 (I) | 8/0.06 (S) | 32/0.5 (I) |
| *A. niger* | | | | | | | | | | |
| AN1 | >64 | 4 | 1 | 0.5 | 1 |  | 32/4 (I) | 32/1 (I) | 32/0.5 (I) | 32/1 (I) |
| AN2 | >64 | 1 | 1 | 1 | 1 |  | 16/1 (I) | 32/1 (I) | 32/1 (I) | 32/1 (I) |
| AN3 | >64 | 4 | 1 | 0.25 | 0.5 |  | 32/4 (I) | 32/1 (I) | 32/0.13 (I) | 32/0.5 (I) |
| AN4 | >64 | 4 | 1 | 0.5 | 2 |  | 32/4 (I) | 32/1 (I) | 64/0.13 (I) | 32/2 (I) |
| AN5 | >64 | 4 | 0.5 | 0.5 | 0.5 |  | 64/2 (I) | 8/1 (I) | 32/0.25 (I) | 32/2 (A) |
| AN6 | >64 | 8 | 1 | 2 | 2 |  | 2/4 (I) | 32/2 (I) | 64/1 (I) | 32/2 (I) |
| AN7 | >64 | 4 | 0.25 | 0.5 | 0.5 |  | 2/2 (I) | 4/0.5 (I) | 2/0.25 (I) | 32/0.5 (I) |
| AN8 | >64 | 2 | 1 | 2 | 2 |  | 8/1 (I) | 32/1 (I) | 32/1 (I) | 32/2 (I) |
| *A. terreus* | | | | | | | | | | |
| 453 | >64 | 2 | 0.25 | 1 | 0.125 |  | 2/1 (I) | 32/1 (A) | 2/0.25 (S) | 8/0.25 (I) |
| 455 | >64 | 2 | 0.5 | 0.5 | 0.5 |  | 32/1 (I) | 8/1 (I) | 2/0.13 (S) | 32/0.5 (I) |
| 465 | >64 | 2 | 0.25 | 1 | 0.25 |  | 64/0.5 (I) | 2/0.5 (I) | 32/0.25 (S) | 32/0.25 (I) |
| 469 | >64 | 4 | 0.5 | 1 | 0.25 |  | 16/2 (I) | 32/0.5 (I) | 16/0.13 (S) | 16/0.5 (I) |
| 476 | >64 | 4 | 0.5 | 1 | 0.5 |  | 16/2 (I) | 32/1 (I) | 8/0.25 (S) | 32/0.5 (I) |
| 479 | >64 | 2 | 0.25 | 1 | 0.125 |  | 64/0.5 (I) | 8/0.5 (I) | 16/0.25 (S) | 16/0.25 (I) |
| 481 | >64 | 4 | 0.5 | 0.5 | 0.5 |  | 32/4 (I) | 4/1 (I) | 64/0.13 (I) | 8/2 (A) |
| 484 | >64 | 4 | 0.5 | 0.25 | 0.5 |  | 32/2 (I) | 4/1 (I) | 16/0.03 (S) | 16/1 (I) |
| 494 | >64 | 4 | 0.5 | 1 | 0.25 |  | 16/2 (I) | 32/0.5 (I) | 8/0.25 (S) | 8/0.5 (I) |
| 498 | >64 | 4 | 1 | 1 | 0.5 |  | 32/4 (I) | 32/1 (I) | 16/0.25 (S) | 32/0.5 (I) |
| 499 | >64 | 4 | 0.5 | 1 | 0.25 |  | 32/2 (I) | 16/1 (I) | 16/0.13 (S) | 8/0.5 (I) |
| 503 | >64 | 2 | 0.5 | 2 | 0.25 |  | 4/1 (I) | 8/1 (I) | 32/0.5 (S) | 32/0.25 (I) |
| 510 | >64 | 4 | 0.5 | 1 | 0.5 |  | 32/2 (I) | 32/0.5 (I) | 16/0.13 (S) | 32/0.5 (I) |
| Quality control | | | | | | | | | | |
| 204304 | >64 | 0.5 | 2 | 0.25 | 2 |  | 32/0.5 (I) | 32/4 (I) | 16/0.06 (S) | 32/2 (I) |
| 3357 | >64 | 0.5 | 2 | 0.125 | 2 |  | 32/0.5 (I) | 32/2 (I) | 8/0.03 (S) | 32/2 (I) |
| a Strains with a yellow background indicate POS‑susceptible strains, while those with a red background denote POS‑resistant strains. The purple, yellow, and red font colors represent S, I, and A, respectively.  b The MIC is the concentration achieving 100% growth inhibition for Aspergillus spp.  c FICI, fractional inhibitory concentration index; S, synergy (FICI of ≤ 0.5); I, no interaction (indifference) (0.5<FICI ≤ 4); A, antagonism (FICI of >4). | | | | | | | | | | |

Table S2 Interaction between UA and azole drugs against Candida spp.

| **Strains^a^** | **MIC^b^ alone (μg/mL)** | | | | |  | **MIC combinations (μg/mL) (FICI^c^)** | | | |
| --- | --- | --- | --- | --- | --- | --- | --- | --- | --- | --- |
|  | UA | ITR | VOR | POS | FLU |  | UA/ITR | UA/VOR | UA/POS | UA/FLU |
| *C. albicans* | | | | | | | | | | |
| 16820 | >32 | 0.125 | 0.5 | 0.125 | 0.25 |  | 16/0.13 (I) | 4/0.13 (S) | 8/0.03 (S) | 16/0.25 (I) |
| 123118 | >32 | 0.0625 | 0.25 | 0.5 | 8 |  | 16/0.06 (I) | 2/0.06 (S) | 8/0.13 (S) | 4/0.25 (S) |
| 122473 | >32 | 0.25 | 4 | 0.5 | 0.25 |  | 32/0.13 (I) | 32/0.06 (I) | 32/0.03 (I) | 16/0.5 (I) |
| 122843 | >32 | 1 | 4 | 0.125 | 0.25 |  | 32/0.06 (I) | 32/0.5 (I) | 4/0.03 (S) | 16/0.25 (I) |
| 129968 | >32 | 1 | 8 | 0.5 | 4 |  | 32/0.13 (I) | 2/2 (S) | 8/0.03 (S) | 16/2 (I) |
| 130110 | >32 | 0.5 | 4 | 0.5 | 2 |  | 8/0.06 (S) | 2/1 (S) | 8/0.03 (S) | 16/0.5 (S) |
| *C. glabrata* | | | | | | | | | | |
| 122127 | >32 | 0.25 | 0.25 | 0.125 | 2 |  | 32/0.06 (I) | 4/0.5 (I) | 32/0.03 (I) | 32/0.06 (I) |
| 121889 | >32 | 0.125 | 0.25 | 0.125 | 2 |  | 16/0.06 (I) | 16/0.5 (I) | 8/0.03 (S) | 16/4 (I) |
| 122512 | >32 | 0.25 | 0.5 | 0.125 | 2 |  | 32/0.06 (I) | 16/0.25 (I) | 32/0.03 (I) | 32/0.06 (I) |
| 58120 | >32 | 0.5 | 4 | 0.25 | 4 |  | 16/0.5 (I) | 32/1 (I) | 16/0.06 (S) | 16/4 (I) |
| 130133 | >32 | 0.25 | 0.25 | 0.25 | 4 |  | 32/0.06 (I) | 16/0.25 (I) | 16/0.06 (S) | 32/0.06 (I) |
| *C.tropicalis* | | | | | | | | | | |
| 122543 | >32 | 0.0625 | 0.0625 | 0.03125 | 0.0625 |  | 16/0.06 (I) | 32/0.25 (A) | 16/0.02 (I) | 16/0.06 (I) |
| 122115 | >32 | 0.25 | 0.5 | 0.03125 | 2 |  | 2/0.13 (I) | 16/2 (A) | 2/0.06 (I) | 4/2 (I) |
| 129221 | >32 | 0.25 | 1 | 0.125 | 1 |  | 16/0.5 (I) | 0.5/2 (I) | 32/0.25 (I) | 16/1 (I) |
| *C. auris* | | | | | | | | | | |
| 381 | >32 | 2 | 0.0625 | 0.25 | No detection |  | 32/2 (I) | 32/0.13 (I) | 1/0.06 (S) | No detection |
| 382 | 32 | 4 | 1 | 0.5 |  |  | 4/1 (S) | 1/0.13 (S) | 1/0.06 (S) |  |
| 383 | 32 | 2 | 0.03125 | 0.03125 |  |  | 4/0.5 (S) | 32/0.03 (I) | 32/0.03 (I) |  |
| 384 | >32 | 4 | 4 | 0.5 |  |  | 8/1 (S) | 16/1 (S) | 1/0.13 (S) |  |
| 385 | >32 | 4 | 4 | 0.25 |  |  | 64/1 (I) | 16/2 (I) | 2/0.06 (S) |  |
| 386 | 32 | 4 | 4 | 0.125 |  |  | 16/0.06 (I) | 16/2 (I) | 8/0.03 (S) |  |
| 387 | 8 | 0.125 | 0.0625 | 0.03125 |  |  | 1/0.06 (I) | 4/0.06 (I) | 4/0.03 (I) |  |
| 388 | >32 | 4 | 1 | 0.5 |  |  | 32/2 (I) | 8/2 (I) | 8/0.13 (S) |  |
| 389 | >32 | 2 | 1 | 0.5 |  |  | 32/2 (I) | 16/0.5 (I) | 1/0.13 (S) |  |
| 390 | >32 | 4 | 0.5 | 0.5 |  |  | 32/2 (I) | 8/2 (A) | 4/0.13 (S) |  |
| Quality control | | | | | | | | | | |
| 22019 | >32 | 4 | 0.0625 | 0.5 | 2 |  | 2/2 (I) | 16/0.06 (I) | 4/0.06 (S) | 16/4 (I) |
| a Strains with a yellow background indicate POS‑susceptible strains, while those with a red background denote POS‑resistant strains. The purple, yellow, and red font colors represent S, I, and A, respectively.  b The MIC is the concentration achieving 100% growth inhibition for Aspergillus spp.  c FICI, fractional inhibitory concentration index; S, synergy (FICI of ≤ 0.5); I, no interaction (indifference) (0.5<FICI ≤ 4); A, antagonism (FICI of >4). | | | | | | | | | | |

Table S3 Interaction between UA and azole drugs against *Exophiala dermatitidis*

| **Strains^a^** | **MIC^b^ alone (μg/mL)** | | | | |  | **MIC combinations (μg/mL) (FICI^c^)** | | | |
| --- | --- | --- | --- | --- | --- | --- | --- | --- | --- | --- |
|  | **UA** | **ITR** | **VOR** | **POS** | **ISA** |  | **UA/ITR** | **UA/VOR** | **UA/POS** | **UA/ISA** |
| *E. dermatitidis* | | | | | | | | | | |
| PYJ1 | >64 | 0.5 | 0.125 | 0.125 | 0.5 |  | 32/0.13 (S) | 32/0.06 (I) | 8/0.03 (S) | 16/0.25 (I) |
| PYJ2 | >64 | 0.5 | 0.25 | 0.25 | 0.5 |  | 64/0.13 (I) | 32/0.25 (I) | 32/0.06 (S) | 32/0.5 (I) |
| PYJ3 | >64 | 0.5 | 0.125 | 0.25 | 0.125 |  | 32/0.13 (S) | 32/0.13 (I) | 16/0.06 (S) | 32/0.13(I) |
| PYJ4 | >64 | 1 | 0.5 | 0.25 | 1 |  | 32/1 (I) | 32/0.5 (I) | 32/0.06 (S) | 32/1(I) |
| PYJ5 | >64 | 1 | 0.5 | 0.5 | 2 |  | 32/1 (I) | 32/0.5 (I) | 8/0.13 (S) | 32/2 (I) |
| PYJ8 | >64 | 1 | 0.125 | 0.125 | 0.25 |  | 16/0.25 (S) | 8/0.06 (I) | 8/0.03 (S) | 32/0.25 (I) |
| PYJ9 | >64 | 1 | 0.25 | 0.25 | 1 |  | 32/0.5 (I) | 32/0.25 (I) | 32/0.03 (S) | 32/1(I) |
| PYJ12 | >64 | 1 | 0.25 | 0.25 | 0.5 |  | 16/0.5 (I) | 32/0.25 (I) | 16/0.06 (S) | 32/0.5(I) |
| PY91 | >64 | 1 | 0.25 | 0.25 | 1 |  | 32/0.5 (I) | 32/0.25 (I) | 32/0.03 (S) | 32/1(I) |
| PY93 | >64 | 2 | 0.5 | 0.25 | 1 |  | 32/2 (I) | 32/0.5 (I) | 32/0.25 (I) | 32/1 (I) |
| PY94 | >64 | 1 | 0.125 | 0,125 | 0.5 |  | 16/0.5 (I) | 32/0.13 (I) | 8/0.03 (S) | 32/0.5 (I) |
| PY95 | >64 | 1 | 0.125 | 0.125 | 0.5 |  | 16/0.5 (I) | 32/0.13 (I) | 16/0.03 (S) | 16/0.25 (I) |
| PY1a | >64 | 1 | 0.25 | 0.125 | 1 |  | 16/0.5 (I) | 32/0.25 (I) | 16/0.03 (S) | 32/1 (I) |
| PY1b | >64 | 2 | 0.5 | 0.5 | 2 |  | 16/1 (I) | 32/0.5 (I) | 16/0.13 (S) | 32/2 (I) |
| Quality control | | | | | | | | | | |
| 204304 | >64 | 1 | 0.5 | 0.25 | 0.25 |  | 8/0.5 (I) | 32/0.5 (I) | 8/0.03 (S) | 32/0.25 (I) |
| a Strains with a yellow background indicate POS‑susceptible strains, while those with a red background denote POS‑resistant strains. The purple, yellow, and red font colors represent S, I, and A, respectively.  b The MIC is the concentration achieving 100% growth inhibition for Aspergillus spp.  c FICI, fractional inhibitory concentration index; S, synergy (FICI of ≤ 0.5); I, no interaction (indifference) (0.5<FICI ≤ 4); A, antagonism (FICI of >4). | | | | | | | | | | |

Table S4 Interaction between UA and azole drugs against *Cryptococcus neoformans*

| **Strains^a^** | **MIC^b^ alone (μg/mL)** | | | | |  | **MIC combinations (μg/mL) (FICI^c^)** | | | |
| --- | --- | --- | --- | --- | --- | --- | --- | --- | --- | --- |
|  | UA | ITR | VOR | POS | FLU |  | UA/ITR | UA/VOR | UA/POS | UA/FLU |
| *C. neoformans* | | | | | | | | | | |
| Y1 | >32 | 2 | 0.0625 | 0.25 | 1 |  | 8/4 (I) | 32/0.25 (A) | 8/0.5 (I) | 16/4 (A) |
| Y2 | >32 | 2 | 0.0625 | 0.25 | 1 |  | 16/4 (I) | 320.25 (A) | 16/0.5 (I) | 8/4 (A) |
| Y3 | >32 | 2 | 0.0625 | 0.25 | 2 |  | 8/4 (I) | 32/0.25 (A) | 8/0.5 (I) | 32/8 (A) |
| Y7 | >32 | 2 | 0.0625 | 0.25 | 1 |  | 8/4 (I) | 16/0.25 (A) | 8/0.5 (I) | 16/4 (A) |
| Y8 | >32 | 2 | 0.0625 | 0.25 | 1 |  | 8/4 (I) | 32/0.25 (A) | 32/1 (A) | 4/2 (I) |
| Y9 | >32 | 1 | 0.0625 | 0.25 | 1 |  | 32/1 (I) | 32/0.25 (A) | 32/0.25 (I) | 16/4 (A) |
| Y11 | >32 | 2 | 0.0625 | 0.25 | 1 |  | 32/2 (I) | 32/0.25 (A) | 8/0.5 (I) | 16/4 (A) |
| Y12 | >32 | 2 | 0.0625 | 0.5 | 1 |  | 8/4 (I) | 32/0.25 (A) | 4/0.5 (I) | 16/4 (A) |
| Quality control | | | | | | | | | | |
| 22019 | >32 | 4 | 0.0625 | 0.5 | 2 |  | 2/2 (I) | 16/0.06 (I) | 4/0.06 (S) | 16/4 (I) |
| a Strains with a yellow background indicate POS‑susceptible strains, while those with a red background denote POS‑resistant strains. The purple, yellow, and red font colors represent S, I, and A, respectively.  b The MIC is the concentration achieving 100% growth inhibition for Aspergillus spp.  c FICI, fractional inhibitory concentration index; S, synergy (FICI of ≤ 0.5); I, no interaction (indifference) (0.5<FICI ≤ 4); A, antagonism (FICI of >4). | | | | | | | | | | |

Table S5. Overall interaction outcomes across all tested isolates

| **Combination** | **n** | **Synergy, n** | **Synergy, %** | **Synergy 95% CI** | **Indifference, n** | **Indifference, %** | **Antagonism, n** | **Antagonism, %** | **Antagonism 95% CI** |
| --- | --- | --- | --- | --- | --- | --- | --- | --- | --- |
| UA+ITR | 110 | 13 | 11.82% | 7.04–19.18% | 9700.00% | 88.18% | 0 | 0 | 0.00–3.37% |
| UA+VOR | 110 | 6 | 5.45% | 2.52–11.39% | 8600.00% | 78.18% | 18 | 16.36% | 10.61–24.39% |
| UA+POS | 110 | 74 | 67.27% | 58.05–75.33% | 3500.00% | 31.82% | 1 | 0.91% | 0.16–4.97% |
| UA+ISA/FLU | 100 | 2 | 2.00% | 0.55–7.00% | 8700.00% | 87.00% | 11 | 11.00% | 6.25–18.63% |

Note: Omnibus Fisher–Freeman–Halton exact tests: synergy P = 2.73 × 10⁻¹¹; antagonism P = 2.82 × 10⁻⁸.

Table S6. Pairwise comparisons among all four combinations

| **Outcome** | **Comparison** | **Difference (percentage points)** | **Lower 95% CI** | **Upper 95% CI** | **Fisher exact P** | **BH-adjusted P** |
| --- | --- | --- | --- | --- | --- | --- |
| Synergy | UA+POS vs UA+ITR | 55.45 | 43.66 | 64.82 | 1.74E-15 | 3.48E-15 |
| Synergy | UA+POS vs UA+VOR | 61.82 | 50.85 | 70.39 | 1.46E-15 | 3.48E-15 |
| Synergy | UA+POS vs UA+ISA/FLU | 65.27 | 54.78 | 73.46 | 7.83E-16 | 3.48E-15 |
| Synergy | UA+ITR vs UA+VOR | 6.36 | -1.26 | 14.28 | 1.48E-01 | 1.78E-01 |
| Synergy | UA+ITR vs UA+ISA/FLU | 9.82 | 2.90 | 17.32 | 6.36E-03 | 9.54E-03 |
| Synergy | UA+VOR vs UA+ISA/FLU | 3.45 | -2.34 | 9.57 | 2.84E-01 | 2.84E-01 |
| Antagonism | UA+POS vs UA+ITR | 0.91 | -2.55 | 4.97 | 1.00E+00 | 1.00E+00 |
| Antagonism | UA+POS vs UA+VOR | -15.45 | -23.51 | -8.41 | 3.87E-05 | 1.16E-04 |
| Antagonism | UA+POS vs UA+ISA/FLU | -10.09 | -17.76 | -3.84 | 1.81E-03 | 2.71E-03 |
| Antagonism | UA+ITR vs UA+VOR | -16.36 | -24.39 | -9.69 | 3.58E-06 | 2.15E-05 |
| Antagonism | UA+ITR vs UA+ISA/FLU | -11.00 | -18.63 | -5.18 | 2.11E-04 | 4.21E-04 |
| Antagonism | UA+VOR vs UA+ISA/FLU | 5.36 | -4.19 | 14.69 | 3.18E-01 | 3.82E-01 |

Table S7. Species-group exact tests

| **Combination** | **Outcome** | ***Aspergillus spp.* (n = 64)** | ***Candida spp.* (n = 24; n = 14 for ISA/FLU)** | ***E. dermatitidis* (n = 14)** | ***C. neoformans* (n = 8)** | **Test** | **P value** | **Interpretation** |
| --- | --- | --- | --- | --- | --- | --- | --- | --- |
| UA+ITR | Synergy | 6/64 | 4/24 | 3/14 | 0/8 | Fisher–Freeman–Halton exact | 3.56E-01 | No significant heterogeneity |
| UA+ITR | Antagonism | 0/64 | 0/24 | 0/14 | 0/8 | Not estimable |  | No antagonism events |
| UA+VOR | Synergy | 0/64 | 6/24 | 0/14 | 0/8 | Fisher–Freeman–Halton exact | 3.92E-04 | Significant heterogeneity |
| UA+VOR | Antagonism | 7/64 | 3/24 | 0/14 | 8/8 | Fisher–Freeman–Halton exact | 2.50E-07 | Significant heterogeneity |
| UA+POS | Synergy | 45/64 | 16/24 | 13/14 | 0/8 | Fisher–Freeman–Halton exact | 9.20E-05 | Significant heterogeneity |
| UA+POS | Antagonism | 0/64 | 0/24 | 0/14 | 1/8 | Fisher–Freeman–Halton exact | 7.27E-02 | No significant heterogeneity |
| UA+ISA/FLU | Synergy | 0/64 | 2/14 | 0/14 | 0/8 | Fisher–Freeman–Halton exact | 4.24E-02 | Significant heterogeneity |
| UA+ISA/FLU | Antagonism | 4/64 | 0/14 | 0/14 | 7/8 | Fisher–Freeman–Halton exact | 5.29E-07 | Significant heterogeneity |
